# Diagnostic criteria and core outcome set development for necrotising otitis externa: the COSNOE Delphi consensus study

**DOI:** 10.1017/S0022215124000513

**Published:** 2024-09

**Authors:** Sirat Lodhi, Kirsty Dodgson, Michael Dykes, Veena Vishwanath, Rohit Bazaz, Sachin Mathur, Glen Watson, Katherine Cartwright, Amy Pearson, Deborah Wearmouth, Richard List, Phillip Yates, Joanna Dixon, Arullendran Puveendran, Margarita Wilson, Katherine Watson, Milo Cullinan, Youssef Mentias, Ruth Capper, Linda Jewes, Sebastian Wallis, David Hamilton, Brook Adams, Mamoona Khalid-Raja, Barzo Faris, Maha Khan, Stefan Linton, Rohma Abrar, Eloise Owen, Vasiliki Bisbinas, Ali Ijaz, Kimberley Lau, Sara Timms, Jack Bruce, Emma Stapleton

**Affiliations:** 1 Department of Otolaryngology, Manchester Royal Infirmary, UK; 2 Department of Microbiology, Manchester Royal Infirmary, UK; 3 Department of Radiology, Manchester Royal Infirmary, UK; 4 Department of Microbiology Wythenshawe Hospital, Manchester, UK; 5 Department of Radiology, Preston Royal Hospital, UK; 6 Department of Otolaryngology, Sheffield Teaching Hospitals, UK; 7 Department of Microbiology, Sheffield Teaching Hospitals, UK; 8 Department of Otolaryngology, Hull University Teaching Hospitals, UK; 9 Department of Microbiology, Hull University Teaching Hospitals, UK; 10 Department of Radiology, Hull University Teaching Hospitals, UK; 11 Department of Otolaryngology, Newcastle Upon Tyne NHS Hospitals, UK; 12 Department of Radiology, Newcastle Upon Tyne NHS Hospitals, UK; 13 Department of Otolaryngology, Sunderland Royal Hospital, UK; 14 Department of Microbiology, Sunderland Royal Hospital, UK; 15 Department of Radiology, Sunderland Royal Infirmary, UK; 16 Department of Otolaryngology, Doncaster Royal Infirmary, UK; 17 Department of Microbiology, Doncaster Royal Infirmary, UK; 18 Department of Otolaryngology, York Hospitals, UK; 19 Department of Microbiology, York Hospitals, UK; 20 Department of Radiology, York Hospitals, UK; 21 Department of Otolaryngology, Stepping Hill Hospital, Stockport, UK; 22 Department of Microbiology, Stepping Hill Hospital, Stockport, UK; 23 Department of Otolaryngology, St John's Hospital, Livingston, UK; 24 Department of Otolaryngology, University of Manchester, UK; 25 Department of Otolaryngology, University of Sheffield, UK; 26 Department of Otolaryngology, Preston Royal Hospital, UK

**Keywords:** Otitis externa, osteomyelitis, diabetes mellitus, immunosuppression therapy

## Abstract

**Objective:**

Evidence for necrotising otitis externa (NOE) diagnosis and management is limited, and outcome reporting is heterogeneous. International best practice guidelines were used to develop consensus diagnostic criteria and a core outcome set (COS).

**Methods:**

The study was pre-registered on the Core Outcome Measures in Effectiveness Trials (COMET) database. Systematic literature review identified candidate items. Patient-centred items were identified via a qualitative study. Items and their definitions were refined by multidisciplinary stakeholders in a two-round Delphi exercise and subsequent consensus meeting.

**Results:**

The final COS incorporates 36 items within 12 themes: Signs and symptoms; Pain; Advanced Disease Indicators; Complications; Survival; Antibiotic regimes and side effects; Patient comorbidities; Non-antibiotic treatments; Patient compliance; Duration and cessation of treatment; Relapse and readmission; Multidisciplinary team management.

Consensus diagnostic criteria include 12 items within 6 themes: Signs and symptoms (oedema, otorrhoea, granulation); Pain (otalgia, nocturnal otalgia); Investigations (microbiology [does not have to be positive], histology [malignancy excluded], positive CT and MRI); Persistent symptoms despite local and/or systemic treatment for at least two weeks; At least one risk factor for impaired immune response; Indicators of advanced disease (not obligatory but mut be reported when present at diagnosis). Stakeholders were unanimous that there is no role for secondary, graded, or optional diagnostic items. The consensus meeting identified themes for future research.

**Conclusion:**

The adoption of consensus-defined diagnostic criteria and COS facilitates standardised research reporting and robust data synthesis. Inclusion of patient and professional perspectives ensures best practice stakeholder engagement.

## Introduction

Necrotising otitis externa is an infective condition of the outer ear, first comprehensively described by Chandler in 1968.^1^ Mainly observed in frail patients with risk factors including advanced age, diabetes and immunosuppression,^2^ its apparent rising incidence^3^ has been attributed to antibiotic resistance,^4,5^ increasing prevalence of diabetes mellitus, an ageing population and enhanced clinician awareness of necrotising otitis externa.^3^

Necrotising otitis externa carries a significant risk of serious complications.^6^ It has a profound impact on patients’ quality of life^7^ and is an increasing burden to health systems.^8^ Despite this, it remains poorly defined, with heterogeneous diagnostic criteria applied across published studies.^9^ Reporting of outcomes is extremely variable,^10^ with no standardisation of the outcomes assessed in published studies.

Diagnostic criteria for necrotising otitis externa were proposed by Cohen *et al*.^11^ in 1987. These are rarely observed in published studies,^9^ likely because they are outdated. Consensus definitions for necrotising otitis externa have recently been published,^12^ but robust, consistent outcome reporting is also needed to optimise future research and reduce heterogeneity in reported outcomes. Standardised diagnostic criteria and core outcome set items are therefore essential.

Core outcome sets are agreed standardised sets of outcomes representing the minimum amount of data that should be measured and reported in all clinical studies of a specific condition.^13^ The validity of a core outcome set depends on robust development, which must include engagement of key stakeholders, including patients, to prioritise outcomes. Internationally accepted best practice for such a consensus study is outlined in the Core Outcome Measures in Effectiveness Trials guidance.^13^ The development of a validated core outcome set improves consistency in outcome reporting,^14^ which facilitates evidence synthesis.

The absence of accepted diagnostic criteria or standardised outcomes for necrotising otitis externa has led to heterogeneity in published studies and subsequently poor evidence to inform clinical management and optimise future research.^9,10,12^ The goals of this study were therefore (1) to follow Core Outcome Measures in Effectiveness Trials guidance^13^ in the development of a core outcome set for necrotising otitis externa using Delphi methodology^15^ with a stakeholder group; (2) to define diagnostic criteria for necrotising otitis externa via stakeholder consensus; and (3) to clarify necrotising otitis externa terminology via stakeholder consensus.

## Methods

**Figure 1.**
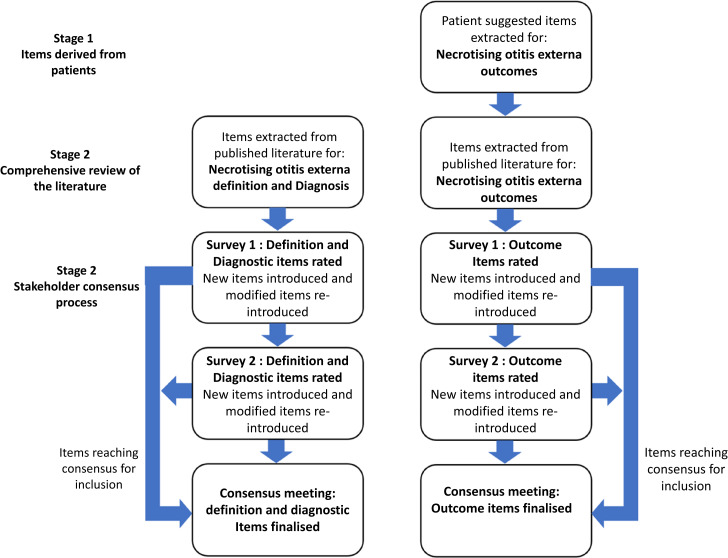
Flowchart of the three-stage process undertaken to complete the Delphi consensus study.

This study was developed based on methodology outlined in the Core Outcome Measures in Effectiveness Trials handbook.^13^ The protocol for this study was registered in the Core Outcome Measures in Effectiveness Trials database (Study 1843). The core outcome set, diagnostic criteria and terminology clarification were developed in parallel. The study was completed in a three-stage process (Figure 1).

A systematic literature review was completed to identify definition, diagnostic and core outcome set items. Patient-suggested items were extracted from our qualitative study exploring patients’ experience of necrotising otitis externa.^7^ Multispecialty stakeholders recruited via our funder's professional network were consulted in a two-stage Delphi process, followed by a consensus meeting, to identify items for inclusion in the terminology, diagnostic criteria and core outcome set for necrotising otitis externa.

### Ethical approval

The Health Research Authority Decision Tool outcome confirmed that National Health Service Research Ethics Committee approval was not required. Written informed consent was obtained from all stakeholders.

### Stage 1: Items derived from patients

A qualitative study was conducted with the specific goal of generating patient-suggested items for the core outcome set. This yielded rich information regarding patient experience of necrotising otitis externa.^7^ The study incorporated the completion of open-ended questionnaires by 16 patients diagnosed with necrotising otitis externa and treated at the supervising author's hospital over a 2-year period, following clinical resolution of their necrotising otitis externa. Four main themes emerged from thematic analysis via a grounded theory approach: severe pain, mental health, quality of life and diagnostic delay. Codes from each theme were extracted for inclusion in the stakeholder consensus process.

### Stage 2: Systematic literature review

A systematic review of the literature was completed by two researchers working independently to identify items for inclusion in the stakeholder consensus process. The Pubmed (9 October 2022, 1945 to 2022) and Embase via Ovid (7 October 2022, 1974 to 2022) databases were searched for all English language studies reporting on necrotising otitis externa.

Studies were de-duplicated and excluded if they were case reports or case series with fewer than 10 patients, conference abstracts, editorials, letters or comments. Abstracts were screened and studies were excluded if they did not address the definition, diagnosis, treatment or outcomes of adults with necrotising otitis externa. Full texts and reference lists were also screened.

Studies were included that incorporated items pertaining to necrotising otitis externa definition, diagnosis, treatment or outcome measures. It was anticipated that studies were likely to report similar items. To minimise duplication, a staged approach was adopted for the extraction of items, based on publication date. Firstly, items were extracted from studies published between 2012 and 2022, followed by studies published between 2010 and 2011. Items published in each extraction period were compared. If new items were identified in the latter extraction period, item extraction from studies published in two-year periods was continued until no further new items were identified. Finally, all extracted items were de-duplicated and categorised into definition, diagnosis or core outcome set categories.

### Stage 3: Stakeholder consensus process

An online survey was created using Google Forms. The survey consisted of all extracted items, divided into three item categories: definition, diagnosis and outcomes. The survey was distributed and analysed in a two-stage Delphi process to identify consensus for each category. Thirty-five stakeholders were identified via the funder's professional network. Stakeholders compromised consultant otologists, microbiologists and radiologists with extensive experience managing patients with necrotising otitis externa in their specialist clinical practice, and students and residents who did not yet have a specialist practice but had previously published on the theme of necrotising otitis externa.

Stakeholders were asked to rate their agreement with the use of one term over another when defining necrotising otitis externa. Diagnostic and core outcome set items were rated on their suitability for inclusion as a minimum diagnostic criterion for a definitive diagnosis of necrotising otitis externa, and suitability for inclusion in a core outcome set for necrotising otitis externa, respectively. Participants were asked to rate statements on a Likert scale from 1 to 5 (1 = strongly disagree, 5 = strongly agree). Free-text responses were invited and were used to guide subsequent rounds.

Based on Core Outcome Measures in Effectiveness Trials guidelines, an item was deemed to have reached consensus and was removed from the process when there was a minimum of 70 per cent stakeholder agreement with a statement and less than 10 per cent disagreement, or vice versa.^16^ Items reaching consensus for inclusion or exclusion were not re-introduced for discussion in subsequent rounds. Items not reaching consensus were re-introduced in round 2 if they were close to reaching consensus or excluded from the two-stage Delphi process in cases where the consensus of stakeholders was neutral. In round 2, new items were introduced in addition to existing items which had been refined and re-introduced based on stakeholder free-text comments.

Items for inclusion in the terminology, diagnostic criteria and core outcome set for necrotising otitis externa were finalised in a consensus meeting with stakeholders.

## Results

### Stage 1: Items derived from patients

Subthemes within each theme were extracted as items (*n* = 11) for inclusion in the core outcome set (Supporting Information File 1). Severe, uncontrolled pain was a significant theme, mentioned by all patients,^7^ along with quality of life, diagnostic delay and the impact of necrotising otitis externa on patients’ mental health, for example ‘I honestly felt suicidal after weeks of terrible pain and very little sleep’.

### Stage 2: Systematic literature review

**Figure 2.**
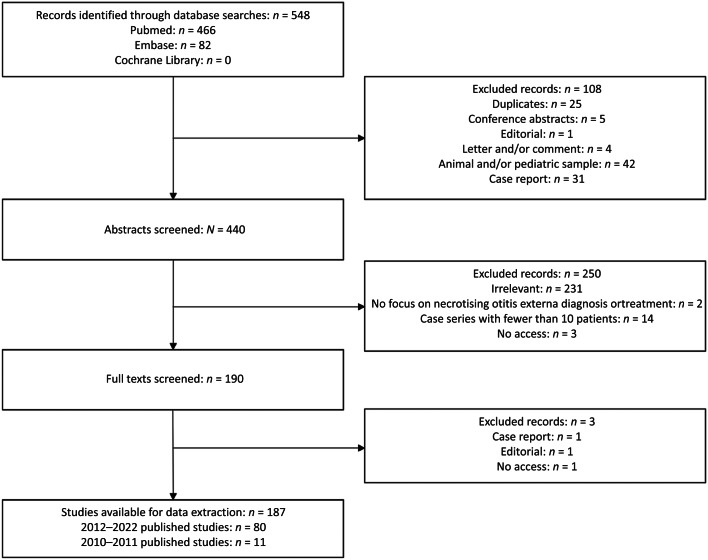
Preferred Reporting Items for Systematic Reviews and Meta-Analysis flowchart of the systematic literature review process.

A search of the Pubmed, Embase and Cochrane Library databases in October 2022 identified 548 articles published since database conception (Figure 2). Abstract and full-text screening resulted in 187 articles that fulfilled the criteria for this study being identified. In the first extraction period, 80 studies were published. In the second extraction period, 11 studies were published. Items were only extracted from the first extraction period because no new items were identified in the second extraction period. In total, 1 definition item, 29 diagnostic items (Supporting Information File 2) and 46 outcome items (Supporting Information File 1) were extracted.

### Stage 3: Stakeholder consensus process

**Figure 3.**
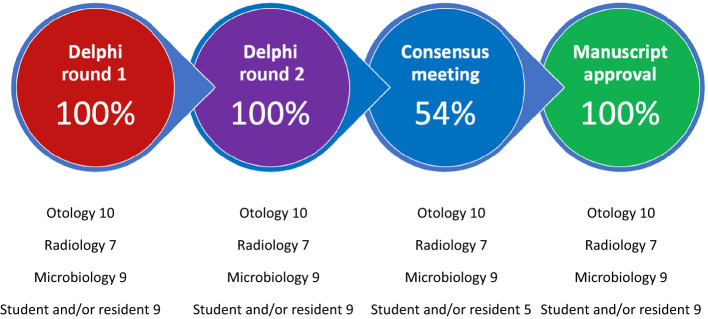
Stakeholder participation in the consensus process.

Thirty-five stakeholders were recruited to the study of 53 invited (66 per cent) and 100 per cent of recruited stakeholders participated in Delphi round 1. In addition, 100 per cent of recruited stakeholders participated in Delphi round 2. Nineteen recruited stakeholders attended the consensus meeting (54 per cent). All stakeholders reviewed and approved the final manuscript (Figure 3). Extracted items were combined and de-duplicated for inclusion in the first Delphi survey (Supporting Information Files 1 and 2). Two Delphi rounds were completed. During each round, stakeholders were asked to complete an online survey. Stakeholders were then invited to participate in an optional consensus meeting to finalise items.

### Necrotising otitis externa terminology

In round 1, consensus was reached favouring the use of the term ‘necrotising’, as opposed to ‘malignant’, when describing necrotising otitis externa. Stakeholders’ free-text comments stated that the use of the term ‘malignant’ may be misinterpreted as cancer and cause patient anxiety.

### Necrotising otitis externa diagnostic criteria

In round 1, 11 of 29 items reached consensus. Of those, one item reached consensus for exclusion and the remainder reached consensus for inclusion. Of the 18 non-consensus reaching items, 8 were re-introduced in round 2 as they were either close to reaching consensus in round 1 or were identified as being a discussion point based on stakeholder free-text comments. This included items referring to raised inflammatory markers (C-reactive protein (CRP), erythrocyte sedimentation rate (ESR), white blood cell (WBC) count and fever).

A ninth item was introduced in round 2: ‘at least one risk factor for immunosuppression’. This item had reached consensus for inclusion in round 1, but was refinement and re-introduced in round 2 based on comments received in round 1. In survey 2, ‘fever’ and ‘at least one risk factor for immunosuppression’ reached consensus for exclusion and inclusion, respectively. Items referring to raised inflammatory markers and ‘positive external auditory canal microbiology’ did not reach consensus, with respondents largely choosing neutrality regarding the inclusion of these items as core diagnostic criteria for necrotising otitis externa.

At the consensus meeting, ‘advanced disease indicators’, a previously non-consensus reaching item, reached consensus for inclusion. Items referring to pain were modified to specify how pain was to be measured. The following measures were added: patient self-reported pain, pain affecting activities of daily living and pain requiring analgesia. In total, 12 items in 6 themes formed the consensus diagnostic criteria for necrotising otitis externa (Table 1).

**Table 1.** COSNOE stakeholder consensus diagnostic criteria for necrotising otitis externa

Theme	Diagnostic item	Consensus definition of item
Signs and symptoms	Oedema Otorrhoea Granulation tissue	EAC swelling Discharge from ear Reactive overhealing in the EAC
Pain	Otalgia Nocturnal otalgia	Can be defined as: self-reported pain and/or pain affecting activities of daily living and/or pain requiring analgesia
Investigations	EAC granulation (microbiology) EAC granulation (histology) Positive computed tomography scan Positive magnetic resonance imaging scan	Positive culture not required but must be reported Biopsy negative for malignancy Bony erosion of ear canal Inflammation extending beyond the ear canal
Duration of symptoms	Persistent symptoms despite local and/or systemic treatment for at least two weeks	
Risk factors	At least one risk factor for impaired immune response	Diabetes mellitus, immunosuppression, immunodeficiency, immunosenescence, frailty, malnutrition
Advanced disease indicators (not obligatory for a diagnosis of necrotising otitis externa but must be reported when present)	Indicators of advanced disease	Cranial nerve palsies Temporomandibular joint involvement Meningitis, cerebral abscess, intracranial thrombosis Extensive radiological change

EAC, external auditory canal

Stakeholders were clear that whilst a minority of immunocompetent patients may present with necrotising otitis externa, the inflammatory response of most necrotising otitis externa patients is impaired because of their immunocompromise, therefore inflammatory marker levels cannot be reliably used to diagnose or monitor necrotising otitis externa. There was a consensus for the inclusion of indicators of advanced disease in the reporting of necrotising otitis externa within consensus diagnostic criteria.

### Necrotising otitis externa outcomes

In round 1, 33 of 50 items reached consensus. Of the 17 non-consensus reaching items, 4 were re-introduced in round 2 as they were close to reaching consensus. Furthermore, one new item was introduced based on round 1 free-text comments: ‘reporting of a co-morbidity index result’. In round 2, two further items reached consensus for inclusion. At the consensus meeting, the non-consensus reaching items ‘improvement in indicators of advanced disease’ and ‘delivery of alternate therapies, e.g. hyperbaric oxygen therapy’, reached consensus for inclusion. This resulted in a total of 36 items within 12 themes reaching consensus for inclusion in the final core outcome set for necrotising otitis externa (Table 2). Stakeholders emphasised the need to define advanced disease and the need to include co-morbidity index scores.

**Table 2.** COSNOE stakeholder consensus core outcome set for necrotising otitis externa

Theme	Core outcome which must be reported
Signs and symptoms	Improvement in oedema and/or swelling Improvement in otorrhoea Resolution of granulation tissue
Pain	Analgesia requirement Improvement in pain (self-reported) Improvement in nocturnal pain (self-reported) Impact of treatment on patient quality of life
Advanced disease indicators	Improvement in indicators of advanced disease
Complications	Cranial nerve dysfunction and resolution Intracerebral abscess diagnosed since commencing treatment Intracerebral thrombus diagnosed since commencing treatment Meningitis diagnosed since commencing treatment Sepsis diagnosed since commencing treatment
Patient survival and death	Death primarily due to necrotising otitis externa and/or its complications Death due to other causes during treatment and follow up Patient survival at one year
Antibiotic regimen and side effects	Antibiotic treatment escalation from oral to intravenous, or de-escalation from intravenous to oral The reason for choice of antibiotic therapy The choice of antimicrobial The reasons for medication changes Any side effects of treatment
Co-morbidities	Validated patient co-morbidity index score
Other treatments	The decision to progress to surgery and rationale for this The delivery of alternate therapies (e.g. hyperbaric oxygen)
Compliance	Patient compliance with treatment Patient compliance with follow up
Duration and cessation of treatment	The duration of total treatment The duration of intravenous antimicrobial treatment The duration of pre-admission out-patient treatment The duration of in-patient stay (include variability due to availability of out-patient antimicrobial therapy) The duration of treatment post discharge The criteria applied for cessation of treatment Post-antibiotic treatment cessation imaging result
Relapse and readmission	The number of patients experiencing necrotising otitis externa relapse (defined as recurrence of ipsilateral necrotising otitis externa in any timescale following cessation of treatment) The number of people requiring hospital re-admission for necrotising otitis externa
Multidisciplinary team management	Whether patients were managed by a multidisciplinary team and details of professionals (e.g. otologist, microbiologist, radiologist etc.)

## Discussion

This consensus study, conducted according to international best practice guidelines,^13^ has achieved its goals, leading to the recommendation of the COSNOE diagnostic criteria for necrotising otitis externa (Table 1) and the COSNOE core outcome set for necrotising otitis externa (Table 2). These are the first of their kind worldwide.

The stakeholder group were consulted regarding whether graded, secondary or optional diagnostic items may be useful. There was very clear consensus on this, with all collaborators agreeing that core, minimum diagnostic criteria are preferred, with no role for graded, secondary or optional items. This is to facilitate the clarity, useability and synthesis of future research.

The third goal of the study, regarding disease terminology, reached consensus in Delphi round 1, with ‘necrotising otitis externa’ clearly preferred over ‘malignant otitis externa’ in keeping with published consensus definition recommendations.^12^ The term ‘malignant’, first introduced by Chandler in 1968,^1^ was thought to incorrectly imply a neoplastic process, risking unnecessary patient anxiety. It is important to note that neither term describes osteomyelitis, which is a key feature of advanced necrotising otitis externa. At our consensus meeting, ‘invasive otitis externa’ was proposed as a more accurate term, but the consensus preference for ‘necrotising otitis externa’ prevailed.

On the theme of terminology, a clear consensus was also achieved in Delphi round 1 regarding necrotising otitis externa ‘relapse’, which respondents defined as recurrence of ipsilateral necrotising otitis externa in any timescale following cessation of treatment (core outcome set item 34, Table 2). Previous work has limited the definition of ‘relapse’ to the three-month period following treatment cessation, but our respondents were unanimous in their consensus that an indefinite timescale better reflects the disease process in necrotising otitis externa.

Our patient-derived items were generated via a qualitative study, which was conducted with the specific goal of generating patient-suggested items for the core outcome set. This yielded rich information regarding patient experience of necrotising otitis externa.^7^ Patients with necrotising otitis externa tend to be elderly, frail patients with medical co-morbidities^17^ and a poor five-year survival rate from both disease-specific and co-morbid causes.^6^ For this reason it was agreed not to include patients in the Delphi process, but to optimise data collection via a parallel qualitative study.^7^ This is a minor departure from Core Outcome Measures in Effectiveness Trials guidelines to accommodate our patient population and may be a useful precedent for core outcome set methodology involving other frail and co-morbid patient populations.

The only previously published diagnostic criteria for necrotising otitis externa date back to 1987,^11^ when Cohen and Friedman proposed minimum diagnostic criteria. Perhaps unsurprisingly, our consensus diagnostic criteria do not depart greatly from these 36-year-old criteria, with both incorporating pain, otorrhoea, oedema, discharge, granulation tissue, positive imaging and immunocompromise.

One of the main discussion points at our consensus meeting was immunosuppression. The wording ‘at least one risk factor for impaired immune response’ (necrotising otitis externa diagnostic criterion 11, Table 1) was agreed to reflect the inclusion of this complex theme. Consensus for the inclusion of risk factors for immunosuppression was observed in all stages of the stakeholder consensus process, despite the publication of rare cases of necrotising otitis externa in apparently immunocompetent individuals.^18^ It was agreed that frailty, immunosenescence and malnutrition should be included as potential risk factors for impaired immune response, alongside diabetes mellitus and other recognised causes of immunosuppression or immunodeficiency. Local risk factors for necrotising otitis externa did not reach consensus for inclusion. Despite the appearance of local risk factors for necrotising otitis externa in the literature,^1,2,19^ they were felt not to be ubiquitous and therefore not a core diagnostic criterion.

Items associated with the immune response to infection were excluded as diagnostic items (fever and adenopathy) or did not reach consensus for inclusion or exclusion (inflammatory markers including CRP, ESR and WBC count). At our consensus meeting, stakeholders agreed on the exclusion of inflammatory marker items as core diagnostic criteria for necrotising otitis externa. It is recognised that individuals with impaired immunity may not present a classic inflammatory response,^20^ hence inflammatory markers cannot be relied on to diagnose necrotising otitis externa because they are non-specific, may be indicative of a non-infectious process and may not be observed during a serious infectious process.^21,22^ Our stakeholder consensus was aligned with this, also adding that as a pseudomonal biofilm infection, necrotising otitis externa can be locally invasive without raising a systemic inflammatory response,^23^ and that absence of systemic inflammatory response may account for delayed necrotising otitis externa diagnoses.^2^ It was agreed that in patients where inflammatory markers are raised in the context of necrotising otitis externa, monitoring them may be a useful marker for response to treatment,^24^ a theme beyond the scope of this study that warrants future research trials.

Pain was another diagnostic theme that elicited discussion. Both otalgia and nocturnal pain reached consensus for inclusion as core diagnostic criteria in round 1. At our consensus meeting, it was agreed that pain as a diagnostic criterion can be self-reported or based on its impact on activities of daily living (e.g. eating, sleep) or on analgesia requirement. Although these measures are subjective, severe pain in necrotising otitis externa is ubiquitous and affects patients’ quality of life.^2^ Recognition of pain and optimisation of analgesia are essential.

Whilst respondents agreed that it is essential to send both solid (granulation) and liquid (pus) samples for microbiological analysis to identify a causative organism, positive microbiology reached consensus for exclusion as a core diagnostic criterion for necrotising otitis externa (necrotising otitis externa diagnostic criterion 6, Table 1). If an organism can be cultured, this guides treatment choices, but negative culture will not preclude the empiric treatment of clinically diagnosed necrotising otitis externa. Culture-negative cases of necrotising otitis externa are well recognised^25^ and may be attributed to prior antibiotic use, commensal microorganisms producing misleading results or fungal disease. Our respondents discussed the role of deep tissue samples and agreed that whilst these can be deployed for refractory cases and to confirm fungal necrotising otitis externa, they can also be unreliable in patients who have had extensive antibiotic therapy.^25^

The delivery of therapies other than antimicrobials was discussed, including surgery and hyperbaric oxygen therapy^26^ for necrotising otitis externa. It was agreed that all interventions for necrotising otitis externa should be reported and ideally published to add to the evidence base.^27^ The reporting of surgical intervention, the decision to progress to surgery and the delivery of any additional or alternative therapies reached consensus for inclusion (core outcome set items 23 and 34, Table 2).

Indicators of advanced disease were identified as an important theme for future research, as they can guide treatment decisions and predict prognosis.^28,29^ Initially, cranial nerve palsy did not reach consensus for inclusion or exclusion as a diagnostic marker of advanced necrotising otitis externa. Based on free-text comments in rounds 1 and 2, it was discussed in our consensus meeting, where it reached consensus for inclusion, not as a core diagnostic criterion per se, but as an essential theme for data reporting within the scope of clinicians making a clinical diagnosis of necrotising otitis externa (necrotising otitis externa diagnostic criterion 12, Table 1).

Respondents agreed that whilst cranial nerve palsies, temporo-mandibular joint involvement and extensive radiological changes can all be markers of advanced disease, there is insufficient evidence to include these as core diagnostic criteria. Heterogeneous clinical and radiological stratification and scoring protocols have been proposed,^30–33^ and none has yet been universally adopted in disease reporting, indicating a lack of robust consensus on this theme.

Notably, positive computed tomography (CT) and magnetic resonance imaging (MRI) scans both reached consensus for inclusion as core diagnostic criteria in round 1 of our consensus process. Imaging recommendations for necrotising otitis externa diagnosis vary across the literature and tend to be based on imaging accessibility and local experience.^34,35^ Whilst widely accessible, bony erosion on CT scanning reflects late disease, with MRI being superior for detection of early disease and for monitoring response to treatment.^32^ More work is required to clarify optimal imaging protocols in necrotising otitis externa, with positron emission tomography-CT becoming an increasingly popular choice^36^ and nuclear imaging declining in popularity, with no single modality of imaging that can provide a complete picture of diagnosis, disease progression and resolution in necrotising otitis externa.^37^

A validated patient co-morbidity index score reached consensus for inclusion (core outcome set item 22, Table 2). It was noted that the Charlson Comorbidity Index has been frequently used in the literature.^38^ Use of a validated score may indicate prognosis and guide clinicians regarding optimal therapy decisions,^39^ and it is also a useful metric for future research synthesis in terms of defining patient populations and examining mortality risk.

### Strengths and limitations

The strengths of this work are that it is a unique and much-needed study in an area of increasing clinical importance in which little evidence exists. The study was conducted according to internationally recognised best practice guidelines^13^ with multidisciplinary stakeholders, using a recognised consensus process.^40^ Stakeholders were free from external influence whilst completing surveys.^41^

We acknowledge that this study type is not without limitations, for example items could be misinterpreted by stakeholders during survey completion. To overcome this, surveys were reviewed carefully by the lead and supervising authors prior to distribution, and were amended and refined based on stakeholder feedback. We have followed our stakeholders’ consensus recommendations, even when these were at odds with our own opinions. The use of a five-point Likert scale offered stakeholders the opportunity to choose neutrality, which led to some rich and powerful discussions at our consensus meeting on themes which may not have been explored in the context of a forced agree or disagree choice.

Our stakeholders were from a variety of multidisciplinary backgrounds and career stages. This approach is recognised for achieving optimal clinical consensus outcomes.^13,14^ Stakeholders were geographically limited to the UK, which may limit the applicability of some recommendations, for example access to imaging modalities may have led to a different outcome in lower income countries or different health systems.

- Necrotising otitis externa is an invasive infective disease with minimal evidence underpinning its diagnosis and management
- The published literature on necrotising otitis externa is low quality with heterogeneous outcome reporting, which impedes data synthesis and prevents robust conclusions and recommendations
- International best practice guidelines from the Core Outcome Measures in Effectiveness Trials initiative were followed to develop consensus diagnostic criteria and a core outcome set for necrotising otitis externa
- The engagement of stakeholders, including patients and multidisciplinary collaborators, ensured best practice for optimal outcomes
- Stakeholders were unanimous that diagnostic criteria should be simple, and that there is no role for optional or secondary diagnostic criteria
- Adoption of the COSNOE diagnostic criteria and core outcome set will facilitate the optimisation of future necrotising otitis externa research through consistency in reporting and enhanced data synthesis, enabling best practice to be identified

We are pleased with our 100 per cent engagement during Delphi rounds 1 and 2, and manuscript approval. The lower engagement rate at our consensus meeting, which was conducted during working hours, reflected the difficulty of balancing clinical and research commitments. We were satisfied that stakeholders unable to attend the consensus meeting were given the opportunity to review the final consensus items.

No diagnostic criteria or core outcome set is perfect. Whilst we feel our goal has been achieved, our recommendations must be implemented judiciously, alongside clinical judgement and practicality. We hope they will standardise and optimise data reporting, facilitating evidence synthesis in future research. We look forward to their refinement in future iterations.

## Conclusion

This study describes the development of standardised diagnostic criteria and a core outcome set for necrotising otitis externa. This has been achieved using international best practice guidelines and incorporating patient and multidisciplinary stakeholder engagement. We hope this will facilitate the optimisation of future necrotising otitis externa research trials through consistency in reporting and enhanced data synthesis, enabling best practice to be identified.

The practice of publishing disparate, heterogeneous studies and case series with inconsistent outcome reporting should be discouraged, as should the recommendation of non-evidence-based disease stratification protocols and guidelines.

This work has highlighted areas for future research that could usefully explore patient experience, indicators of advanced disease, clarity regarding imaging, disease monitoring, and treatment modification and cessation. As further work is completed, we hope the diagnostic criteria and core outcome set proposed might be refined further.

## Supporting information

Lodhi et al. supplementary material Lodhi et al. supplementary material

## Supplementary material

The supplementary material for this article can be found at https://doi.org/10.1017/S0022215124000513.

Primary data are available as supplemental files.
