# Supplementary material for: Diagnostic criteria and core outcome set development for necrotising otitis externa: the COSNOE Delphi consensus study
Source: J Laryngol Otol. 2024 Apr 22;138(9):913–20. doi: 10.1017/S0022215124000513 (PMC11518678; doi:10.1017/S0022215124000513)
Supplement: Lodhi et al. supplementary material [file S0022215124000513sup001.docx]

**Supplemental file 1. Summary of COS items derived from patients and systematic literature review, included in the Delphi survey**

Abbreviations: CRP, C reactive protein; ESR, erythrocyte sedimentation rate; HbA1c, glycated haemoglobin; NOE, necrotising otitis externa; WCC, white blood cell count

| **Literature review outcome item(s)** | **Patient suggested outcome item(s)** |
| --- | --- |
| • Death due to NOE  • Death due to non NOE  • On patient survival (6 months, 1 year, 2 years, 5 years)  • On improvement in discharge from ear/otorrhoea  • On improvements in external auditory canal inflammation  • On external auditory canal swelling/ oedema  • On improvements in reactive over healing in ear canal (granulations)  • On improvements in hearing impairment  • On cranial nerve dysfunction  • On intracerebral abscess diagnosed since commencing treatment  • On intracerebral thrombus diagnosed since commencing treatment  • On meningitis diagnosed since commencing treatment  • On sepsis diagnosed since commencing treatment  • On the need for analgesia  • On improvement in ear pain (otalgia) based on patient self reported pain  • On improvement in nocturnal otalgia based on self reporting  • On improvement in headache based on self reported pain  • On impact on quality of life (eg patients said sleep, symptoms impacting life, impact on family and loved ones) as being an important theme for patients in encrotiting otitis externa management  • On compliance with treatment  • On compliance with follow up  • On ability to tolerate imaging  • On the need for antibiotic treatment escalation from roal to IV,or de escalation from IV to oral  • On th reason for choice of antibiotic therapy  • On the choice of antimicrobial  • On the reasons for medication changes  • On the need for surgery  • On the delivery of alternate therapes eg hyperbaric oxygen therapy  • On duration of total treatment  • On duration of IV antibiotic treatment  • On duration of pre admission Op treatment  • On duration of IP stay  • On duration of treatment post discharge  • On criteria for cessation of treatment  • On the post antibiotic treatment termination imaging result  • On imaging at 1 year follow up result  • On pre admission treatment delays  • On treatment delays in secondary care  • On number f people lost to follow up  • On number of people experiencing necrotising otitis external relapse i.e recurrence of ipsilateral necrotising otitis externa in any timescale following cessation of treatment  • On the number of people requiring hospital re admission for necrotising otitis externa post discharge  • On side effects of treatment  • On stabilisation of CRP post treatment  • On stabilisation of ESR post treatment  • On stabilisation of WCC post treatment  • On stabilisation of HbA1c post treatment  • On whether patients were managed by a Multidisciplinary Team eg otologist, microbiologist, radiologist | • On the need for analgesia  • On improvement in ear pain (otalgia) based on patient self reported pain  • On improvement in nocturnal otalgia based on self reporting  • On improvement in headache based on self reported pain  • On improvement in jaw/ tmj pain based on self reported pain (patient reported only)  • On improvement in pain on eating based on patient self reported pain (patient reported only)  • On improvement in pai elsewhere I the body based on self reported pain (patient reported only)  • On impact on quality of life (eg patients said sleep, symptoms impacting life, impact on family and loved ones) as being an important theme for patients in necrotising otitis externa management  • On impact on mental health (patients highlighted mental health eg depression, low mood, wanting to die, anxiety about potential relapse) as being important theme for NOE management  • On pre admission treatment delays  • On treatment delays in secondary care |

**Supplemental file 2: Summary of definition and diagnosis items derived from the systematic literature review, included in the Delphi survey**

| **Outcome category** | **Literature review item(s)** |
| --- | --- |
| **Definition** | - Malignant vs necrotising |
| **Diagnosis** | - EAC swelling/oedema - Discharge (otorrhoea) - Reactive over healing (granulations) - Ear canal erythema - Micro abscess when operated - Hearing impairment - Ear pain (otalgia) - Nocturnal otalgia - Pain behind ear/ mastoid pain - Jaw TMJ pain or jaw locking/trismus - Headache - Facial pain - Fever - Adenopathy - Positive EAC microbiology - EAC granulation biopsy for histo and micro - Deep tissue biopsy - Positive CT scan - Positive MRI scan - Additional imaging (pls specify) - Facial nerve palsy or lower cranial nerve palsy - Raised crp - Raised esr - Raised wcc - Poor glycaemic control (hba1c) - Persistent symptoms despite treatment for at least 2 weeks (local or systemic) - At least one local risk factor listed below:   -Water exposure to external auditory canal  -Humid, warm climate  -External auditory canal trauma  -Ear syringing  -Previous surgery  -Previous radiotherapy  -External auditory canal cholesteatoma   - At least one risk factor for immunosuppression listed below   -Diabetes mellitus  -Old age (immunosenescence)  -Immunosuppression or immunodeficiency  -Frailty  -Malnutrition   - At least one medical comorbidity (heart/lung/liver/kidney disease) |

Abbreviations: CT, computerised tomography; CRP, C reactive protein; ESR, erythrocyte sedimentation rate; HbA1c, glycated haemoglobin; MRI, magnetic resonance imaging; WCC, white blood cell count
